# Oxidative stress in adolescents with overweight/obesity

**DOI:** 10.5937/jomb0-59562

**Published:** 2026-01-06

**Authors:** Marija Bozovic, Barbara Ostanek, Jelena Kotur-Stevuljević, Janja Marc, Filiz Mercantepe, Aleksandra Klisic

**Affiliations:** 1 Institute of Public Health of Montenegro, Podgorica, Montenegro; 2 University of Ljubljana, Faculty of Pharmacy, Department of Clinical Biochemistry, Ljubljana, Slovenia; 3 University of Belgrade, Faculty of Pharmacy, Department for Medical Biochemistry, Belgrade; 4 Recep Tayyip Erdogan University, Faculty of Medicine, Department of Endocrinology and Metabolism, Rize, Turkey; 5 University of Montenegro, Faculty of Medicine, Podgorica, Montenegro; 6 Center for Laboratory Diagnostics, Primary Health Care Centre, Podgorica, Montenegro

**Keywords:** antioxidants, cardiovascular risk, inflammation, obesity, oxidative stress, antioksidansi, kardiovaskularni rizik, inflamacija, gojaznost, oksidativni stres

## Abstract

**Background:**

The pathophysiological mechanism underlying obesity and related diseases is still incompletely understood. A small number of studies employed sophisticated statistical techniques, such as principal component analysis (PCA), to investigate the relationship between oxidative stress, cardiometabolic biomarkers, and obesity in the adolescent population. Hence, we aimed to examine this relationship.

**Methods:**

A total of 68 adolescents (i.e., 34 were overweight/obese, and 34 were sexand age-matched normal-weight controls) were included in the study. Total oxidant status (TOS) and total antioxidant status (TAS) were measured, whereas their ratios were calculated, i.e., pro-oxidant score [(TOS/TAS)*100] and antioxidant score (TAS/TOS). PCA was applied to reduce the number of determined data by grouping them into factors.

**Results:**

A significantly higher concentration of TAS, TOS, and their pro-oxidant ratio (TOS/TAS)*100, while the antioxidant score of TAS/TOS was considerably lower in overweight/obese adolescents compared to normal-weight peers. TOS was the most significant predictor of obesity status (P= 0.001). PCA extracted 3 factors related to obesity status: Factor 1 (gender, creatinine, uric acid, total bilirubin, TAS, waist circumference, and urea), Factor 2 (ALT and AST), and Factor 3 (age, glucose, total protein, and TOS). Among them, Factor 2 (P= 0.003) and Factor 3 (P= 0.003) were independently associated with obesity.

**Conclusions:**

The present study provides evidence of disrupted redox homeostasis in adolescents with obesity. Obesity is tightly connected with increased oxidative stress and a cluster of metabolic abnormalities. It is essential to identify risk factors promptly and develop a strategy to combat obesity and its associated diseases.

## Abstract (sr)

**Uvod:**

Patofizioloski mehanizmi koji leže u osnovi gojaznosti i s njom povezanih bolesti jos uvek nisu u potpunosti razjašnjeni. Mali broj studija koristio je sofisticirane statističke metode, poput analize glavnih komponenti (PCA), kako bi ispitao povezanost između oksidativnog stresa, kardiometaboličkih biomarkera i gojaznosti kod adolescenata. Zbog toga je cilj naše studije bio da istraži ovu povezanost.

**Metode:**

U studiju je ukupno bilo uključeno 68 adolescenata, od kojih je 34 imalo prekomernu telesnu težinu/gojaznost, dok je 34 činilo kontrolnu grupu adolescenata sa normalnom telesnom težinom, usklađenih po polu i starosti. Mereni su ukupni oksidativni status (TOS) i ukupni antioksidativni status (TAS), a na osnovu njih su izračunati prooksidativni skor [(TOS/TAS) x 100] i antioksidativni skor (TAS/TOS). Za redukciju broja ispitivanih varijabli i njihovo grupisanje u faktore primenjena je analiza glavnih komponenti (PCA).

**Rezultati:**

Uočena je značajno veća koncentracija TAS, TOS i njihov prooksidativni odnos (TOS/TAS)*100, dok je antioksidativni skor TAS/TOS bio značajno niži kod pregojaznih/gojaznih adolescenata u poređenju sa vršnjacima normalne težine. TOS je bio najznačajniji prediktor statusa gojaznosti (P= 0,001). PCA je izdvojila 3 faktora povezana sa statusom gojaznosti: Faktor 1 (pol, kreatinin, mokraćna kiselina, ukupni bilirubin, TAS, obim struka i urea), Faktor 2 (ALT i AST) i Faktor 3 (starost, glukoza, ukupni proteini i TOS). Među njima, Faktor 2 (P = 0,003) i Faktor 3 (P= 0,003) su bili nezavisno povezani sa gojaznošću.

**Zaključak:**

Ova studija pruža dokaze o narušenoj redoks homeostazi kod adolescenata sa gojaznošću. Gojaznost je usko povezana sa povišenim oksidativnim stresom i klasterom metaboličkih poremećaja. Pravovremeno prepoznavanje faktora rizika i razvoj efikasnih strategija za prevenciju i lečenje gojaznosti i pratećih oboljenja od suštinskog su značaja.

## Introduction

Childhood obesity is one of the major public health challenges due to its rapid increase in prevalence and associated comorbidities [1]. The prevalence of obesity in children and adolescents aged 5-19 years globally has quadrupled in the last 40 years [1]. Data indicate that every third child in Europe is obese [2]. About 75-80% of adolescents with obesity will become adults with obesity [3]. A sedentary lifestyle, characterised by a lack of physical activity and unhealthy dietary patterns, including the consumption of fast food and sweet beverages, are the major contributors to the increased prevalence of childhood obesity [3].

Although cardiovascular diseases (CVD) rarely manifest in childhood, the risk exists in even overweight/obese young populations [4] [5] [6]. Moreover, children with obesity are at increased risk of type 2 diabetes and non-alcoholic fatty liver disease, both independent risk factors for CVD later in life [7].

Adipose tissue, especially visceral, is a significant source of various mediators of inflammation and pro-oxidants [8]. Oxidative stress, characterised by an imbalance between increased production of reactive oxygen species (ROS) and reactive nitrogen species (RNS) and reduced antioxidant protection, is at the root of obesity and its related diseases [8] [9]. ROS/RNS are extremely reactive and lead to structural and functional damage to almost all tissues and organs, including lipid peroxidation, oxidative modification of proteins, and damage to DNA structures [3] [9]. ROS/RNS have a short half-life and are difficult to measure. That is why it is essential to determine their secondary products in a timely manner before irreversible cell damage occurs [10].

There is a wide variety of ROS/RNS secondary products, and many of them have been investigated in the population with obesity and obesity-related cardiometabolic disorders [8] [9] [10] [11]. However, studies have yielded conflicting results, and none of these markers have been demonstrated to be sufficiently specific and sensitive for routine diagnostics [10] [12].

Given the fact that pro-oxidants interact synergistically, there is a need for a more reliable indicator of redox imbalance that would encompass all pro-oxidants in one assay. Total oxidant status (TOS) is a comprehensive indicator of all prooxidants. It may, therefore, represent a more reliable indicator of the degree of oxidative damage than any single pro-oxidant biomarker [13]. On the other hand, the level of oxidative stress depends on the degree of antioxidant protection that includes non-enzymatic (i.e., obtained via supplements and diet or endogenous biomolecules) and enzymatic antioxidants that neutralise/diminish the harmful effects of ROS/RNS [14]. Total antioxidant status (TAS) reflects a measure of all antioxidants in one assay [14], whereas the TOS/TAS ratio (so-called oxidative stress index, OSI) and TAS/TOS ratio represent comprehensive parameters of overall oxidative stress and antioxidant defence, respectively [12] [13] [14].

However, the pathophysiological mechanism underlying obesity and related diseases is still incompletely understood. Additionally, a small number of studies employed sophisticated statistical techniques, such as principal component analysis (PCA), to investigate the intricate relationship between oxidative stress, cardiometabolic biomarkers, and obesity in adolescent populations. Since studies examining biomarkers of oxidative stress have yielded conflicting results [11] [15] [16], the aim of this research is to comprehensively investigate the levels of oxidative stress and antioxidant defence in adolescents with overweight or obesity.

## Materials and methods

### Study population

The present study included a total of 68 adolescents from two secondary schools in Podgorica. Among them, 34 were overweight/obese, and 34 were sex- and age-matched normal-weight controls. The Institutional Ethics Committee approved the study protocol, and the research was conducted in accordance with the principles of the Declaration of Helsinki. Each participant provided signed informed consent. Additionally, for adolescents under 18 years of age, written permission from their parents was also obtained. The adolescents were asked to fill in a questionnaire regarding medication use, illnesses, and lifestyle habits (i.e., alcohol use and smoking). Besides willingness to participate, the inclusion criteria were normal weight or overweight/obese adolescents who were otherwise healthy and aged 16-19 years. Those adolescents with a history of smoking, alcohol consumption, comorbidities, signs of infection, and high sensitivity C-reactive protein (hsCRP) 10 mg/L were excluded from the study.

Adolescents underwent anthropometric measurements for body weight, body height, and waist circumference (WC) before venipuncture, and their body mass index (BMI) was calculated. Adolescents were considered to be normal weight if presented with a BMI<25 kg/m^2^, whereas those greater than or equal to 25 kg/m^2^ were considered to be overweight/obese.

### Biochemical analyses

The venipuncture was done in the morning after an overnight fast of at least 8 hours. Serum levels of glucose, hsCRP triglycerides (TG), total cholesterol (TC), high-density lipoprotein cholesterol (HDL-c), low-density lipoprotein cholesterol (LDL-c), alanine aminotransferase (ALT), aspartate aminotransferase (AST), total proteins, uric acid, total and direct bilirubin, urea, and creatinine were measured by a Roche Cobas c503 chemistry analyser (Roche Diagnostics GmbH, Mannheim, Germany). ABTS as a chromogen was applied for the determination of TAS [13]. TOS was measured with o-dianisidine [14].

### Statistical analysis

The distribution of data was checked using the Shapiro-Wilk test (for groups with ≤ 50 subjects), taking into account that each group had 34 subjects. The Mann-Whiney U test was used to compare the differences between two groups of unrelated data, while the Kruskal-Wallis test was used to compare 3 or more groups of data. The Wilcoxon paired test was used to compare related data (pairs). Correlation analysis was performed using Spearman's non-parametric correlation analysis. Predictors of body nutrition were assessed using binary logistic regression analysis. Factor analysis was applied as a principal component analysis (PCA) with the aim of grouping data (variables) according to equal variability and reducing the number of variables by grouping them into factors. Multiple linear regression analysis was used to estimate the best predictor model of certain parameters of interest. ROC analysis (receiver operating characteristic curve) was used to check the diagnostic accuracy of the parameters.

For all statistical tests, the basic criterion for the existence of statistical significance is that the obtained P in a two-sided test was ≤0.05 (specified level of significance α).

## Results

The subjects of this study were divided into two groups: normal-weight and overweight/obese subjects. Both groups consisted of adolescents of similar age (median age 16 and 17 years, respectively) and had the same gender distribution (32% boys and 68% girls in each group). These data confirm the adequate selection of subjects in the two study groups.

Basic biochemical parameter comparison determined in this study showed a significantly higher concentration of the inflammatory marker hsCRP in overweight/obese subjects, as well as significantly higher activity of the enzyme ALT However, the values of both parameters were in the reference range (Table 1). Lipid status parameters showed no statistically significant difference between the two investigated groups.

**Table 1:** Basic clinical and sociodemographic data of study subjects. BMI - body mass index; P - Mann-Whitney U test

Parameter	Normal-weight adolescents<br>(n = 34)	Overweight/obese adolescents<br>(n = 34)	P
Age (years)	16 (16-18)	17 (16-18)	0.155
Gender (m/f, n (%))	11/23 (32/68)	11/23 (32/68)	0.602
BMI (kg/m^2^)	21.4 (20.2-22.7)	26.0 (25.5-26.2)	<0.001
WC (cm)	72.0 (68.0-77.0)	82.0 (78.0-86.0)	<0.001
Glucose (mmol/L)	4.60 (4.50-4.90)	4.55 (4.40-4.80)	0.217
Urea (mmol/L)	3.95 (3.40-4.50)	3.85 (3.10-4.50)	0.716
Creatinine (μmol/L)	62.5 (56.0-74.0)	61.5 (57.0-74.0)	0.885
Uric acid (μmol/L)	246 (209-293)	285.5 (228.0-321.0)	0.114
hsCRP (mg/L)	0.40 (0.30-0.60)	1.05 (0.30-2.80)	0.001
Total proteins (g/L)	77 (75-79)	76 (73-79)	0.177
Total bilirubin (μmol/L)	9.3 (5.9-12.7)	9.6 (6.8-15.8)	0.652
Bilirubin, direct (μmol/L)	4.3 (2.9-6.2)	4.2 (3.1-7.1)	0.600
AST (U/L)	20 (16-21)	20 (18-23)	0.187
ALT (U/L)	14 (12-19)	19 (14-27)	0.002
TC (mmol/L)	3.95 (3.51-4.35)	3.93 (3.57-4.32)	0.639
HDL-c (mmol/L)	1.43 (1.27 -1.61)	1.42 (1.30-1.68)	0.665
LDL-c (mmol/L)	2.14 (1.83-2.47)	2.03 (1.79-2.35)	0.864
TG (mmol/L)	0.78 (0.66-0.87)	0.72 (0.57-0.92)	0.927
TG/HDL-c ratio	0.52 (0.41-0.65)	0.49 (0.38-0.68)	0.732

**Figure 1:**
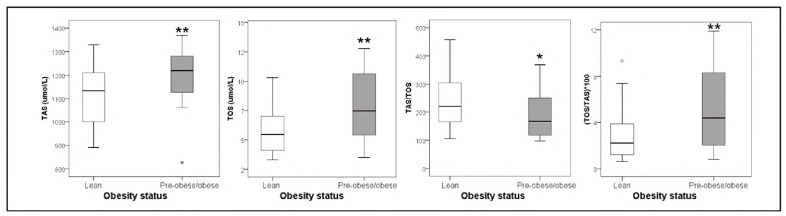
Parameters of redox status (oxidative stress and antioxidant protection) of study subjects. *, ** P < 0.05, 0.01 vs. lean subjects

Redox status parameters are shown in Figure 1. Analysis of this part of the results reveals a significantly higher concentration of TAS, TOS, and their prooxidant ratio (TOS/TAS)*100, whereas the antioxidant score of TAS/TOS was significantly lower in overweight/obese adolescents compared to their normal-weight peers.

Using binary logistic regression analysis (univariate model), we examined the potential of the predictors identified in this study to determine obesity status. Statistical data from this analysis related to significant predictors of nutritional status (belonging to the group of overweight/obese adolescents) are shown in Table 2.

**Table 2:** Binary logistic regression analysis of predictors of nutritional status. SE - standard error, OR - odds ratio (95th CI - confidence interval); P from binary logistic regression analysis

Parameter	B (SE)	Wald coefficient	OR (95% CI)	P
ALT	0.074 (0.035)	4.4	1.08 (1.00-1.16)	0.036
hsCRP	1.78 (0.665)	7.2	5.96 (1.62-21.9)	0.007
TAS	0.006 (0.002)	7.9	1.006 (1.002-1.011)	0.005
TOS	0.395 (0.121)	10.8	1.48 (1.17-1.88)	0.001
TAS/TOS	-0.007 (0.003)	5.54	0.993 (0.987-0.999)	0.019
OSI=(TOS/TAS)*100	0.301 (0.101)	8.9	1.35 (1.109-1.647)	0.003

Significant predictors of obesity status were hsCRP, TAS, and TOS, as well as the TAS/TOS and TOS/TAS indices, with TOS being the most significant predictor (P=0.001).

ROC (receiver operating characteristic curve) analysis was used to check the diagnostic potential (accuracy) of the parameters for assessing the nutritional status of the body. The obtained results are shown in Table 3.

**Table 3:** ROC analysis: the diagnostic accuracy of biomarkers for assessing obesity status. AUC - area under the curve, 95% CI - confidence interval, SE - standard error

Parameter	AUC (95% CI)	SE	P
ALT	0.730 (0.607-0.853)	0.063	0.002
hsCRP	0.730 (0.599-0.861)	0.067	0.002
TAS	0.719 (0.597-0.841)	0.062	0.002
TOS	0.732 (0.614-0.850)	0.060	0.001
OSI=(TOS/TAS)*100	0.707 (0.584-0.830)	0.063	0.003

According to the results of this analysis, the parameter with the highest diagnostic accuracy is the TOS.

Multiple linear regression analysis was used to determine which parameters are the most significant predictors of TAS and TOS values, respectively. In this research, the best model for TAS prediction consisted of the following variables: gender, uric acid, total protein, HDL-c, and waist circumference. The model shows more than 50% influence on TAS concentration (adjusted R^2^=0.545), and uric acid is the most significant predictor (P=0.003).

Predictors of TOS concentration were determined using multiple linear regression analysis, and gender, urea, and WC were included in the best model, with urea and WC being the most significant in the model. This model shows a 21% impact on the TOS value. The results are shown in Table 4.

**Table 4:** Multiple linear regression analysis for defining predictors of TAS and TOS values. CI - confidence interval, SE - standard error

Parameters for TAS prediction <br>(adj. R_2_= 0.545)	B (95% CI)	SE	P
Gender	-64.3 (-136.5-7.9)	36.1	0.080
Uric acid	0.920 (0.332-1.5)	0.294	0.003
Total proteins	5.3 (-0.802-11.5)	3.1	0.087
HDL-c	135.6 (41.4-229.7)	47.0	0.006
WC	4.5 (0.816-8.3)	1.9	0.018
Parameters for TOS prediction<br>(adj. R_2_= 0.211)	B (95% CI)	SE	P
Gender	1.319 (-0.178-2.817)	0.749	0.083
Urea	1.101 (0.384-1.819)	0.359	0.003
WC	0.137 (0.050-0.224)	0.043	0.003

Factorial analysis, specifically Principal Component Analysis (PCA), was applied to reduce the number of parameters determined in this study. The adequacy of the factor analysis was proven by the Kaiser-Meyer-Olkin (KMO) index of 0.586 (the condition is greater than 0.500) and Bartlett's sphericity index (P<0.001, the condition is P<0.05). The detailed results of the factor analysis (PCA with vari- max rotation) are presented in Table 5. The study revealed a total percentage of variability of 47%, with Factor 1 accounting for 23%, Factor 2 for 13%, and Factor 3 for 11%. Factor 1 included gender, creatinine, uric acid, total bilirubin, TAS, WC, and urea. Factor 2 included ALT and AST. Factor 3 included age, glucose, total protein, and TOS.

**Table 5:** Factorial analysis in the group of normal-weight and overweight/obese adolescents.

Factor	Variables	Factor loadings	Variability percentage<br>(total 47%)
Factor 1	Gender	-0.820	23
	Creatinine	0.818	
	Uric acid	0.794	
	Total bilirubin	0.688	
	TAS	0.654	
	WC	0.589	
	Urea	0.571	
Factor 2	ALT	0.913	13
	AST	0.849	
Factor 3	Age	0.710	11
	Glucose	-0.594	
	Total proteins	-0.553	
	TOS	0.516	

The factorial analysis enables the extraction of factors from several different parameters that are similar to each other in terms of variability level and, simultaneously, the formation of scores (numerical values) from those factors. This further enables the factor analysis scores thus obtained to be included in other statistical analyses and comparisons. In this case, these three newly formed parameters are included in a binary logistic regression analysis, which will examine the potential predictive value of the factors extracted by factorial analysis (PCA). The results are shown in Table 6.

**Table 6:** Binary logistic regression analysis of predictors of obesity status. SE - standard error, OR - odds ratio (95^th^ CI - confidence interval)

Parameter	B (SE)	Wald coefficient	OR (95% CI)	P
Factor 1	0.292 (0.256)	1.3	1.339 (0.811-2.209)	0.254
Factor 2	1.696 (0.566)	9.0	5.452 (1.798-16.538)	0.003
Factor 3	1.021 (0.344)	8.8	2.776 (1.415-5.447)	0.003

Binary logistic regression analysis revealed that the second and third factors are significant predictors of obesity status, i.e., the combined effect of the parameters included in these factors (ALT, AST, age, glucose, total protein, and TOS).

## Discussion

The results of the current study indicate both an increased level of oxidative stress and an increased level of antioxidant protection in overweight or obese adolescents compared with their normal-weight peers.

Previous studies have examined the level of oxidative stress in young populations, but they have yielded conflicting results [11] [15] [16]. Moreover, previous studies on redox homeostasis were conducted in younger populations than ours [3] [17] [18]. Rowicka et al. [3] investigated the oxidative stress status in prepubertal children (aged 2-10 years). They found higher total oxidant capacity (TOC) and oxidative stress index (OSI) but lower total antioxidant capacity (TAC), in children with obesity. Kilic et al. [18] found higher TAS and TOS in children with obesity (aged 6-16) but no difference in the TOS/TAS ratio.

Adipose tissue is a significant source of ROS that affects insulin signalling pathways. The diminished activity of phosphatidylinositol 3-kinase (PI3K) and, at the same time, the enhanced activity of Janus kinase (JAK) and protein kinase Cd (PKC-d) in adipocytes [19] contribute to inflammation, insulin resistance, platelet aggregation, vasoconstriction, and endothelial dysfunction, thus promoting cardiometabolic disorders related to obesity [10] [20] [21]. Insulin resistance also favours the increased lipolysis in adipose tissue, thus enhancing the hepatic flux of free fatty acids. Increased liver fat peroxidation, oxidative phosphorylation, and lipogenesis contribute to liver steatosis and atherogenic dyslipidemia [22].

In the present study, factorial analysis (PCA) was employed to further investigate the pathophysiological characteristics of obesity in a young population. PCA enabled the identification of several key contributing factors; among them, the factor consisting of transaminases (AST and ALT) and the factor that clustered age, glucose, total protein, and TOS were significant predictors of obesity status, which underscores the complex interplay between different signalling pathways in obesity.

Cardiometabolic risk factors often coexist and can exert a joint influence on endothelial dysfunction [23]. Indeed, in our previous study [10], we demonstrated that the additive effects of obesity (i.e., BMI 30 kg/m^2^) and oxidative stress increased the probability of a higher cardiovascular risk score in postmenopausal women. Namely, an increase in TOS or OSI by 1 unit doubled the probability of higher cardiovascular risk. By inclusion of obesity status (i.e., BMI 30 kg/m2), the probability of higher cardiovascular risk increased by more than 8 times [10].

On the other hand, there is a wide range of antioxidants which act in a fine-tuned and synergistic manner. The determination of the activity of each one by one is lengthy and complex and may not be an indicator of the overall antioxidant imbalance. The components of TAS include thiol groups, reduced glutathione, uric acid, bilirubin, vitamin E, vitamin C, vitamin A, proteins, etc. [10] [14]. Hence, TAS may be an early sign of alteration of biomolecules and a reliable marker of the overall antioxidant imbalance [12]. The present study shows higher TAS in overweight/obese adolescents, which is in line with the results of Colak et al. [24], who also recorded higher TAS in youngsters with increased CVD risk compared to the controls and opposite to Rowicka et al. [3], who showed lower TAC in children with obesity.

The discrepancy of results can be explained by the different methodologies of measuring the parameters of redox homeostasis, but also by differences in the age of the subjects, the presence of comorbidities, and the use of drugs, all of which can additionally affect the level of oxidative stress [10] [11] [23] [25]. We also assume that one of the causes of this discrepancy in the results is the duration of obesity [3]. In young obese people whose obesity status did not last long enough to manifest obesity-related cardiometabolic disorders, the enzymes of antioxidant protection might not be depleted yet. In the present study, no differences were observed in the levels of glucose and lipid parameters between overweight/obese and normal-weight 16-19-year-old adolescents. Regarding antioxidants, unlike bilirubin, the level of uric acid showed a trend of increasing in overweight/obese adolescents; however, it did not reach statistical significance. This suggests that antioxidants can counteract the increased formation of ROS/RNS, and an increase in TAS may serve as a compensatory mechanism to combat oxidative stress. In the case of prolonged obesity and the presence of associated diseases, it is expected that the enzymes involved in antioxidant protection will be depleted [3] [12].

Recent findings clearly indicate that the prevention of childhood obesity should begin in primary school, encompassing both adherence to healthy dietary patterns, such as a Mediterranean diet rich in nutrients with antioxidant and anti-inflammatory properties, and increased awareness among parents of the necessity of adopting a healthy lifestyle for their children [26].

The study's limitations include its cross-sectional nature and the small number of participants, which are similar to those in previous studies [15] [16] [17] [18]. The lack of physical activity data and dietary survey data, which may affect redox homeostasis [26], is another drawback, as is the absence of fat tissue quantification using precise diagnostic methods such as computed tomography (CT) and magnetic resonance imaging (MRI) [27] [28]. However, the use of CT is not recommended in children due to its significant radiation burden. In contrast, MRI is expensive and, hence, not used in routine diagnostic protocol for fat tissue quantification [29]. On the other hand, the study's strength lies in its comprehensive use of oxidative stress and antioxidant protection parameters, rather than relying on any individual biomarker, taking into account their mutual effects. Thus, we have gained a more precise insight into the redox imbalance in overweight/obese young individuals. Moreover, we have included a relatively narrow age range of adolescents in the research, thereby excluding hormonal variations associated with puberty versus prepuberty. Importantly, all participants were nonsmokers and did not use any medical therapy [23], which reduced the influence of confounding factors on redox balance. The presented results refer to the adolescent population in Montenegro and are not applicable to the other ethnic groups. Therefore, multiethnic and multicenter studies are necessary to confirm our results. Future studies with a larger number of subjects and with a longitudinal design would expand the research mentioned above and provide better insight into redox imbalance in the long-term follow-up of overweight/obese adolescents.

## Conclusions

The present study provides evidence of disrupted redox homeostasis in adolescents with obesity. Obesity is tightly connected with increased oxidative stress and a cluster of metabolic abnormalities. These findings underscore the urgent need to develop a strategy to cope with obesity and diminish oxidative stress, both through changes in healthier dietary patterns that include a diet rich in dietary antioxidants as well as by increasing physical activity to prevent the adverse effects of obesity later in life.

## Dodatak

### Acknowledgements

This work was financially supported in part by a grant from the Ministry of Education, Science and Innovation, Montenegro (project number 01-082/22-1681/3), and by the Ministry of Science, Technological Development and Innovation, the Republic of Serbia through two Grant Agreements with University of Belgrade-Faculty of Pharmacy No 451-03-136/2025-03/ 200161 and No 451-03-137/2025-03/ 200161, and the Slovenian Research Agency, Slovenia (research program P3-0298, projects J3-4527, BI-ME/23-24-030 and CEEPUS network SI-0611, award No. CPNR: M-SI-0611-2324-177307.

The authors would like to thank Anika Murovec for technical assistance in the measurement of selected oxidative stress parameters.

### Conflict of interest statement

All the authors declare that they have no conflict of interest in this work.
